# Advancing Osteoarthritis Treatment: The Therapeutic Potential of Mesenchymal Stem Cell-Derived Exosomes and Biomaterial Integration

**DOI:** 10.3390/biomedicines12112478

**Published:** 2024-10-28

**Authors:** Chung-Hua Chu, Ru-Ping Lee, Wen-Tien Wu, Ing-Ho Chen, Kuang-Ting Yeh, Chen-Chie Wang

**Affiliations:** 1 Department of Orthopedic Surgery, Taipei Tzu Chi Hospital, Buddhist Tzu Chi Medical Foundation, New Taipei City 231016, Taiwan; tch19832@tzuchi.com.tw; 2 Department of Biomedical Engineering, National Taiwan University, Taipei 106216, Taiwan; 3 Institute of Medical Sciences, Tzu Chi University, Hualien 970374, Taiwan; fish@gms.tcu.edu.tw (R.-P.L.); timwu@tzuchi.com.tw (W.-T.W.); 4 Department of Orthopedics, Hualien Tzu Chi Hospital, Buddhist Tzu Chi Medical Foundation, Hualien 970473, Taiwan; ihchen@tzuchi.com.tw; 5 School of Medicine, Tzu Chi University, Hualien 970374, Taiwan; 6 Graduate Institute of Clinical Pharmacy, Tzu Chi University, Hualien 970374, Taiwan

**Keywords:** exosomes, osteoarthritis, mesenchymal stem cells, biomaterials, cartilage regeneration

## Abstract

Background/Objectives: Osteoarthritis (OA) is a prevalent and debilitating joint disorder characterized by progressive cartilage degradation and inflammation, for which traditional treatments offer only symptomatic relief without halting disease progression. Exosomes, cell-free vesicles derived from mesenchymal stem cells, have emerged as a promising alternative therapy owing to their regenerative and anti-inflammatory properties. Methods: This review synthesizes findings from recent studies (2017–2023) on the therapeutic potential of exosomes in OA treatment, highlighting their ability to modulate the joint microenvironment, reduce inflammation, and promote cartilage repair by delivering bioactive molecules such as cytokines, growth factors, and regulatory ribonucleic acids. Results: We explore the integration of exosomes with biomaterials, such as hydrogels and scaffolds, to enhance their delivery and therapeutic efficacy, and we address the critical challenges associated with their clinical application, including standardization of isolation and characterization methods, scalability of production, mechanistic understanding, and long-term safety. Despite these challenges, exosome-based therapies offer several advantages over traditional and cell-based treatments, including lower immunogenicity, ease of handling, and targeted delivery of therapeutic agents to damaged tissues. Conclusions: We provide an analytical perspective on the current state of exosome research in OA, emphasizing the need for standardized production methods, deeper mechanistic insights, and rigorous long-term safety assessments. Future directions should focus on optimizing delivery systems, exploring personalized medicine approaches, and conducting comparative effectiveness studies to fully realize the potential of exosome therapies for OA treatment. Addressing these gaps will be crucial for translating exosome therapies from bench to bedside and achieving a transformative impact on OA management.

## 1. Introduction

Osteoarthritis (OA) is a chronic, degenerative joint disorder characterized by the progressive breakdown of multiple joint tissues, including articular cartilage, the synovial membrane, subchondral bone, menisci, and the infrapatellar fat pad (IFP) [1]. It leads to pain, stiffness, and functional limitations, primarily affecting weight-bearing joints such as the knees and hips, as well as smaller joints of the hands [2]. OA is the most prevalent form of arthritis, impacting millions globally, and its prevalence continues to rise due to the aging population and increasing obesity rates [3,4].

Traditionally, OA has been viewed primarily as a disease of cartilage; however, it is now recognized as a whole-joint disease that affects various joint tissues. Articular cartilage, the most commonly affected tissue, undergoes progressive degradation as an imbalance between anabolic and catabolic processes leads to the loss of the smooth articular surface [5]. This degradation exposes the underlying subchondral bone, causing pain and joint dysfunction [1]. The synovial membrane also plays a significant role in OA progression by becoming inflamed and producing pro-inflammatory cytokines such as interleukin-1 beta (IL-1β) and tumor necrosis factor-alpha (TNF-α). These cytokines exacerbate cartilage degradation and contribute to the overall inflammatory state within the joint [6]. Subchondral bone remodeling is another critical component of OA, resulting in sclerosis, osteophyte formation, and altered load distribution. These changes further worsen joint stiffness and pain [3]. Additionally, the menisci, which are essential for joint stability and cushioning, often deteriorate in OA, leading to further instability, cartilage loss, and compromised joint function [1,4]. The development and progression of OA are influenced by several risk factors. Age is a significant contributor, as joint tissues naturally degenerate over time, making older individuals more susceptible [7]. Obesity is another crucial factor, as it not only increases the mechanical load on joints but also promotes systemic inflammation, accelerating disease progression [8]. Previous joint injuries or repetitive stress can also lead to the early onset of OA, as they compromise the integrity of joint structures, including cartilage and menisci [9]. Genetic predisposition is an additional factor, as certain gene variants have been associated with an increased risk of developing OA [10]. Recent studies have highlighted the critical role of the IFP in OA pathology [11,12,13,14]. The IFP, an adipose tissue structure located beneath the patella, is closely associated with the synovial membrane, forming an anatomical-functional unit [11]. In OA, the IFP becomes inflamed and fibrotic, releasing pro-inflammatory cytokines and adipokines that contribute to joint inflammation and pain [12]. Changes in the biomechanical properties of the IFP also exacerbate joint dysfunction [13]. The interaction between the IFP and synovium amplifies inflammation within the joint, further contributing to cartilage degradation [14]. Understanding the role of the IFP as a key modulator of OA pathology provides insights into comprehensive therapeutic strategies that target not just the cartilage but also the inflammatory processes involving the IFP and synovium [13]. 

Traditional treatments for OA primarily focus on symptom management using pharmacological interventions, such as nonsteroidal anti-inflammatory drugs (NSAIDs) and intra-articular corticosteroid injections [15]. While these treatments provide temporary relief, they do not halt disease progression or regenerate damaged tissues [16]. In recent years, regenerative medicine has highlighted the potential of mesenchymal stem cells (MSCs) for OA treatment due to their ability to differentiate into various cell types and secrete paracrine factors that modulate inflammation and promote tissue repair [17,18]. Despite these promising advances, challenges such as cellular senescence, the potential for malignant transformation, and variability in treatment outcomes remain significant barriers to the clinical application of MSC therapy [19,20].

Exosomes, which are small extracellular vesicles (EVs) derived from MSCs, have emerged as a novel therapeutic option that can overcome some of the limitations associated with direct stem cell therapies [21,22]. According to the Minimal Information for Studies of Extracellular Vesicles (MISEV2023) guidelines, exosomes are recognized for their significant role in intercellular communication and therapeutic applications [23]. These vesicles transport proteins, lipids, and nucleic acids that influence the behavior of recipient cells, thereby promoting anti-inflammatory effects and tissue regeneration [24,25]. Exosome-based therapies offer distinct advantages, including lower immunogenicity, ease of storage and handling, and the ability to deliver bioactive molecules directly to specific joint tissues, such as cartilage, synovium, and subchondral bone [26,27].

Furthermore, the integration of exosomes with biomaterials has emerged as a promising strategy for enhancing therapeutic efficacy. Biomaterials provide a protective environment for exosomes, facilitating their controlled release and maintaining their stability and bioactivity at the site of injury [28]. This approach ensures sustained delivery, improving the potential for cartilage regeneration and overall joint health. The use of such biomaterial-assisted exosome therapies represents a frontier in OA treatment, offering not only symptom relief but also the potential for significant regeneration of damaged joint tissues.

This review aims to explore the current understanding of exosome biology, its application in OA, and future directions for clinical translation. Between 2017 and 2023, eight key studies highlighted the regenerative properties and anti-inflammatory effects of exosomes derived from MSCs and other sources [16,20,21,22,25,27,28,29,30,31,32,33,34] (Table 1). Additionally, these studies discussed the integration of exosomes with biomaterials to enhance delivery and efficacy, presenting a promising approach for developing advanced therapies for OA.

## 2. Biological Properties and Mechanisms of Exosomes

Exosomes are a subset of EVs with diameters ranging from 30 to 150 nm, secreted by most cell types, including MSCs. According to the MISEV 2023 guidelines, exosomes play a critical role in intercellular communication by transferring bioactive molecules, such as proteins, lipids, and various forms of ribonucleic acids (RNAs), including messenger RNA (mRNA) and microRNA (miRNA) [23]. This communication is essential in maintaining tissue homeostasis and modulating cellular responses under both physiological and pathological conditions [35].

### 2.1. Biogenesis of Exosomes

**Figure 1:**
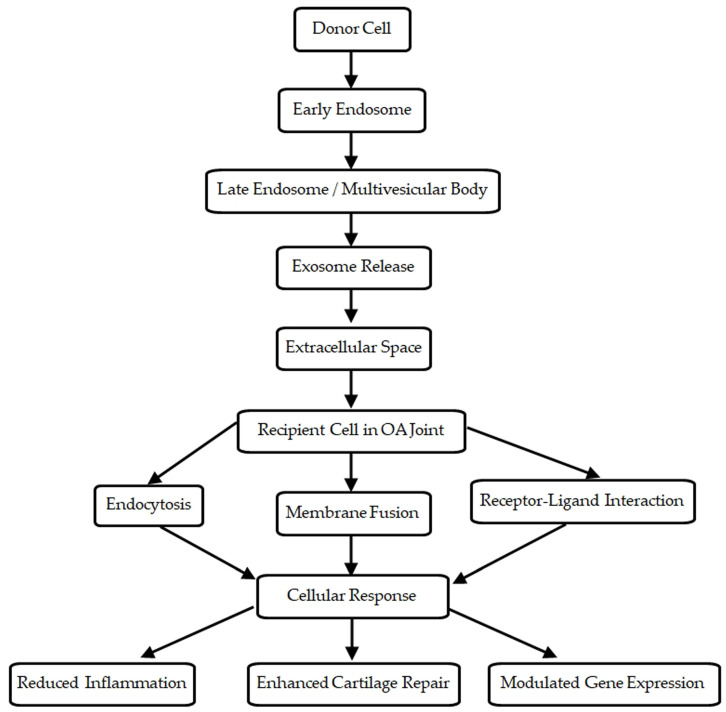
Exosome biogenesis and mechanism of action in osteoarthritis. This figure shows the process of exosome formation from a donor cell, their release into the extracellular space, and their various mechanisms of interaction with recipient cells in the OA joint.

The biogenesis of exosomes and their mechanism of action in OA are illustrated in Figure 1. The process is initiated through the inward budding of the plasma membrane, which results in the formation of early endosomes. These early endosomes mature into late endosomes, accumulating intraluminal vesicles (ILVs) via the inward budding of their membranes. The resulting multivesicular bodies either fuse with lysosomes for degradation or with the plasma membrane, releasing the ILVs as exosomes into the extracellular environment. The release of exosomes is regulated by the endosomal sorting complexes required for transport (ESCRT) pathway, although ESCRT-independent pathways also play a role in exosome formation [16].

The content and surface markers of exosomes are highly dependent on the cells from which they originate. Exosomal cargo reflects the molecular composition of their parent cells, which makes them ideal candidates for both therapeutic applications and as diagnostic biomarkers. Exosomes contain a complex cargo that includes proteins, lipids, RNAs, and deoxyribonucleic acid fragments, which can interact with recipient cells to modulate various biological processes, including inflammation, tissue regeneration, and immune responses [27].

### 2.2. Exosome Cargo and Its Functional Implications

One of the unique features of exosomes is their ability to carry and protect a diverse range of biomolecules within their lipid bilayers. The lipid bilayer of exosomes shields their cargo from enzymatic degradation, ensuring efficient delivery to target cells. Upon uptake by recipient cells, this cargo can modulate cellular activities by influencing gene expression, altering signaling pathways, and reshaping the cellular microenvironment.

#### 2.2.1. Proteins

Exosomes are enriched with proteins that reflect the physiological state of their parent cells. These include membrane-bound proteins, such as tetraspanins (cluster of differentiation [CD] 9, CD63, CD81), which play roles in exosome formation and uptake by recipient cells. Exosomes also contain cytoskeletal proteins, enzymes, and heat shock proteins, which can contribute to tissue repair processes [23]. Notably, MSC-derived exosomes are rich in anti-inflammatory cytokines and growth factors, such as transforming growth factor-beta (TGF-β) and bone morphogenetic proteins (BMPs), which promote cartilage repair and reduce inflammation in OA [24].

#### 2.2.2. Ribonucleic Acids

Exosomes also carry various forms of RNA, including mRNA and miRNA, which play significant roles in gene regulation. MSC-derived exosomes contain miRNAs, such as miR-140 and miR-21, which downregulate the expression of pro-inflammatory genes and matrix-degrading enzymes, thus contributing to the preservation of cartilage in patients with OA [16]. The presence of mRNAs within exosomes can lead to the synthesis of specific proteins in recipient cells, further influencing their behavior. For example, mRNA encoding for anti-inflammatory proteins can be translated in recipient cells to counteract inflammation.

#### 2.2.3. Lipids

The lipid bilayer of exosomes provides structural integrity and contributes to their functional properties. Exosomal membranes are rich in sphingomyelins, cholesterol, and phosphatidylserine, which facilitate fusion with recipient cells and play roles in immune modulation. These lipids can interact with cell surface receptors, leading to the activation of signaling pathways that regulate cell survival, proliferation, and apoptosis [26].

### 2.3. Mechanisms of Intercellular Communication

Exosomes exert their biological effects primarily through three mechanisms: direct fusion with the recipient cell membrane, receptor–ligand interactions, and endocytosis. Upon fusion or endocytosis, the exosomal cargo is delivered to the cytoplasm of recipient cells, where it influences cellular processes.

MSC-derived exosomes are particularly potent in modulating the immune system and promoting tissue repair. Through receptor–ligand interactions, exosomes can bind to specific receptors on target cells, triggering signaling cascades that lead to anti-inflammatory and regenerative responses. For example, exosomes carrying TGF-β can bind to TGF-β receptors on chondrocytes, promoting cartilage repair and reducing inflammation in the joint microenvironment of patients with OA [23].

Furthermore, exosomes can alter gene expression in recipient cells by transferring miRNAs and mRNAs. The miRNAs contained within exosomes can suppress pro-inflammatory gene expression by targeting specific mRNA transcripts for degradation or translational repression. This is particularly relevant in OA, where MSC-derived exosomes downregulate the expression of matrix metalloproteinases (MMPs) and other catabolic enzymes that contribute to cartilage degradation [28].

### 2.4. Therapeutic Potential in OA

The biological properties of MSC-derived exosomes make them an attractive therapeutic tool for treating OA. Their ability to deliver a cargo of anti-inflammatory cytokines, growth factors, and regulatory RNAs directly to damaged tissues enables them to modulate the joint microenvironment and promote cartilage regeneration. Exosome therapy can reduce inflammation, enhance cartilage repair, and prevent further degradation of joint tissues in OA models [27].

MSC-derived exosomes are particularly effective in reducing inflammation by delivering anti-inflammatory proteins and miRNAs to the synovium and cartilage. For example, miR-140 within exosomes specifically downregulates the expression of Matrix metallopeptidase (MMP)-13, a key enzyme responsible for cartilage breakdown in OA. Additionally, growth factors, such as BMPs, promote the proliferation of chondrocytes and the synthesis of extracellular matrix (ECM) components, such as collagen and aggrecan, which are essential for maintaining cartilage integrity [28].

By preventing apoptosis and promoting the survival of chondrocytes, exosomes can halt the progression of OA and facilitate the repair of damaged cartilage [36]. This regenerative potential, coupled with their low immunogenicity, makes exosomes a promising alternative to cell-based therapies, which carry risks of immune rejection and tumorigenicity [29].

## 3. Therapeutic Potential of Exosomes in Osteoarthritis

The ability of exosomes to reduce inflammation, prevent cartilage degradation, and promote regeneration underscores their therapeutic potential in OA. Compared with traditional treatments, such as NSAIDs and corticosteroids, which only provide symptomatic relief, exosome-based therapies target the root causes of OA by modulating the joint microenvironment [27]. MSC-derived exosomes offer regenerative benefits, addressing both inflammation and tissue repair, making them a superior alternative to conventional therapies.

### 3.1. Anti-Inflammatory Effects

Exosomes play a pivotal role in modulating the inflammatory response in OA [37]. Chronic inflammation is a hallmark of OA, leading to the overexpression of matrix-degrading enzymes, such as MMP-13, and cytokines, such as tumor necrosis factor-α [16]. MSC-derived exosomes contain miRNAs and anti-inflammatory cytokines that suppress these pro-inflammatory pathways, creating a more favorable environment for tissue repair [38]. By targeting the underlying inflammatory mechanisms of OA, exosomes offer a more holistic approach to disease management [39].

### 3.2. Cartilage Regeneration and Repair

Exosomes also promote cartilage repair by delivering bioactive molecules that enhance chondrocyte proliferation and ECM synthesis [40]. Growth factors, such as TGF-β and BMPs, stimulate the anabolic activity of chondrocytes, leading to the production of essential cartilage components, such as collagen and aggrecan [28]. This regenerative capacity helps prevent further cartilage degradation, addressing the degenerative aspect of OA directly.

### 3.3. Advantages over Traditional and Stem Cell Therapies

Exosomes have several advantages over both traditional and cell-based therapies. Their low immunogenicity reduces the risk of immune rejection, a common concern with stem cell therapies [29]. Additionally, exosomes are easier to store, transport, and administer compared with living cells. They are acellular and therefore pose no risk of tumorigenicity, making them safer for long-term use [41].

## 4. Integration of Exosome Therapy with Biomaterials for Enhanced Delivery

**Figure 2:**
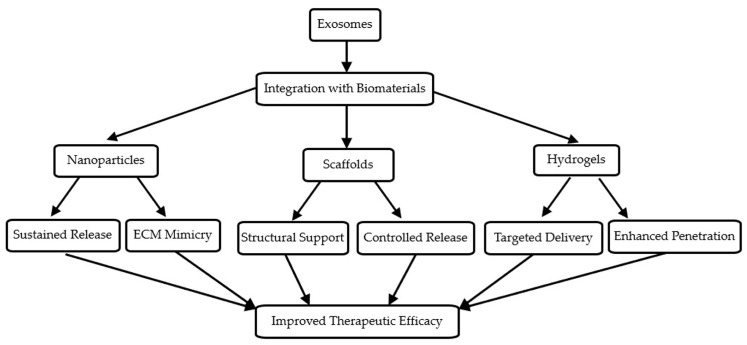
Exosome biogenesis and mechanism of action in osteoarthritis. This figure provides a visual comparison between traditional OA treatments and exosome-based therapy, highlighting the key advantages of exosome-based approaches.

Although exosomes show great promise in OA treatment, one major challenge is their rapid clearance from the joint space, limiting their therapeutic efficacy. Integrating exosome therapy with biomaterials, such as hydrogels, scaffolds, and nanoparticles, can improve exosome stability, retention, and controlled release, enhancing their therapeutic outcomes [30]. The integration of exosomes with the various biomaterials for enhanced delivery and therapeutic efficacy is illustrated in Figure 2. This approach combines the regenerative potential of exosomes with the controlled release and structural support provided by biomaterials.

### 4.1. Hydrogels and Scaffolds for Exosome Delivery

Hydrogels are highly biocompatible materials that mimic the natural ECM of the cartilage, making them ideal for delivering exosomes in OA treatment. By encapsulating exosomes, hydrogels allow for a sustained and localized release at the injury site, preventing their rapid clearance from the joint cavity [28]. However, scaffolds provide structural support for tissue regeneration while releasing exosomes in a controlled manner. Scaffolds made from materials, such as collagen and poly (lactic-co-glycolic acid), improve cartilage repair in preclinical models [31].

### 4.2. Nanoparticles for Targeted Delivery

Nanoparticles are another promising platform for exosome delivery. Their small size allows for deeper tissue penetration and improved targeting of damaged cartilage. Functionalized nanoparticles can enhance the specificity of exosome delivery, ensuring that exosomes reach their intended target without off-target effects. Additionally, nanoparticles protect exosomes from enzymatic degradation, prolonging their therapeutic activity in the joint space [28].

### 4.3. Controlled Release and Bioavailability

A Biomaterial-based delivery systems may provide controlled release of exosomes in response to specific stimuli, such as changes in pH or enzymatic activity in inflamed joints [42]. This ensures that exosomes are released at the right time and place, maximizing their therapeutic efficacy while minimizing the risk of side effects. For example, enzyme-responsive scaffolds release exosomes in response to MMPs, which are upregulated in OA joints [32].

## 5. Challenges in the Clinical Application of Exosome-Based Therapies

Although exosome-based therapies hold significant promise for the treatment of OA, several challenges must be addressed to fully realize their therapeutic potential. These challenges include issues related to the isolation, characterization, scalability, and regulatory aspects of exosome production and the need for a deeper understanding of their mechanisms of action and long-term safety [43].

### 5.1. Standardization of Exosome Isolation and Characterization

One primary challenge in exosome therapy is the standardization of exosome isolation and purification methods. Current techniques, such as ultracentrifugation, size exclusion chromatography, and immunoaffinity capture, vary in efficiency, purity, and scalability. These variations can affect the quality and reproducibility of exosome preparations, thereby affecting their therapeutic efficacy and safety [27]. Standardizing these methods is crucial for advancing exosome therapy from the laboratory to clinical settings. Recent studies have highlighted the potential of bioengineering approaches to improve exosome production and function [31,32,44]. For example, bioengineering exosomes by modifying their surface markers or encapsulating them in hydrogels can enhance their targeting ability, retention, and therapeutic efficacy [31,32].

### 5.2. Scalability of Exosome Production

The scalability of exosome production is a significant hurdle. Producing clinical-grade exosomes in sufficient quantities for widespread therapeutic use requires highly controlled, scalable, and cost-effective manufacturing. Current production methods are typically labor-intensive and have low yields, which are not conducive to large-scale production [45]. Therefore, developing bioreactor-based culture systems and other industrial-scale production techniques is critical for meeting clinical demands [26]. Bioengineered exosomes have shown promise in enhancing production efficiency, and recent studies have explored novel techniques for improving both the quality and quantity of exosome yields in a scalable manner [31,44,46].

### 5.3. Regulatory Challenges

From a regulatory perspective, exosome therapies face unique challenges owing to their classification as biological products and potential drug delivery systems [47]. Regulatory frameworks specific to exosome therapies are still under development, and guidelines for clinical trials, quality control, and therapeutic use must be established [48]. Future studies should focus on overcoming these challenges through technological innovation and collaborative research. Furthermore, the potential for using exosomes in personalized medicine for OA treatment is being explored, suggesting that regulatory frameworks will also need to accommodate patient-specific exosome therapies [34].

## 6. Future Directions and Opportunities for Exosome-Based Therapies

### 6.1. Mechanistic Understanding

Another challenge is the need for a deeper understanding of exosomes’ mechanisms of action. Owing to their nanoscale size and complex cargo, it is challenging to comprehensively characterize exosomal contents and their biological activities. Advanced analytical techniques are required to profile the proteins, lipids, and nucleic acids within exosomes and understand how these components contribute to their therapeutic effects [23]. This detailed understanding is essential for identifying the most therapeutically potent exosome subpopulations and developing targeted therapies. The exosomal cargo, particularly the microRNAs and proteins they carry, are key modulators in promoting cartilage repair and mitigating inflammatory responses in OA [34,35].

### 6.2. Bioengineering and Integration with Biomaterials

In addition to bioengineering exosomes for improved production and efficacy, integrating exosomes with biomaterials is a promising strategy to enhance their delivery and therapeutic potential. Biomaterials, such as hydrogels, scaffolds, and nanoparticles, can encapsulate exosomes and provide a controlled release at the site of injury. This approach maximizes the stability and bioavailability of exosomes, improving their regenerative and anti-inflammatory effects [32]. Future studies should explore the most effective combinations of biomaterials and bioengineered exosomes to optimize therapeutic outcomes.

### 6.3. Long-Term Safety and Personalized Medicine

Although MSC-derived exosomes have demonstrated favorable safety profiles in initial studies, long-term safety concerns remain a key consideration [49]. Potential risks include immunogenicity, tumorigenicity, and off-target effects, given the ability of exosomes to modulate cellular activity [50]. It is essential to conduct comprehensive safety evaluations, particularly in the context of immune responses and potential malignancy [51]. As exosome-based therapies advance toward clinical applications, ensuring the safety and consistency of exosome preparations through rigorous testing will be vital [32,35]. Furthermore, exploring the potential for personalized medicine in exosome therapy will be crucial, as tailored treatments can provide more effective and patient-specific outcomes [34].

## 7. Discussion

**Figure 3:**
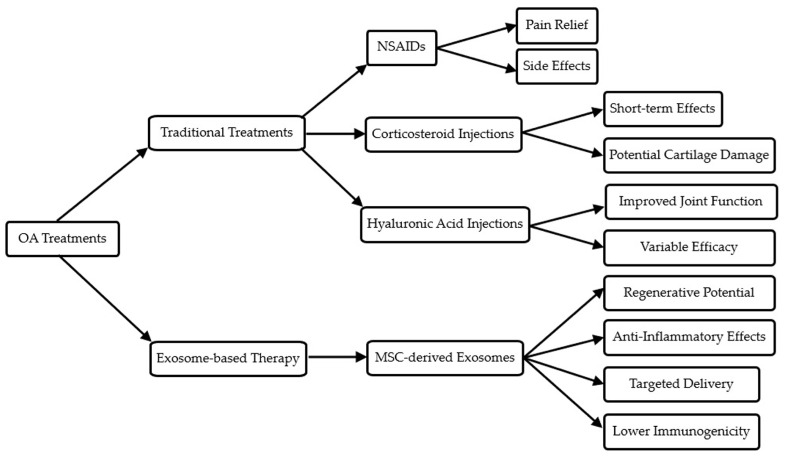
Integration of exosomes with biomaterials for enhanced delivery. This figure demonstrates how exosomes can be integrated with various biomaterials (hydrogels, scaffolds, and nanoparticles) to enhance their delivery and therapeutic efficacy in OA treatment.

This review explored the emerging role of MSC-derived exosomes in the treatment of OA, underscoring their potential to modulate the joint microenvironment, reduce inflammation, and promote cartilage repair. The use of exosomes, particularly MSC-derived exosomes, in the treatment of OA offers a groundbreaking approach that extends beyond the limitations of traditional therapies. Although conventional treatments, such as NSAIDs and corticosteroids, focus on providing symptomatic relief, they fail to address the underlying causes of OA or promote cartilage repair [15]. By contrast, exosome-based therapies hold regenerative potential, as exosomes carry a cargo of bioactive molecules, including growth factors, cytokines, and miRNAs, which can modulate the joint microenvironment, reduce inflammation, and stimulate tissue regeneration [27,29]. This section aims to discuss the significant advantages, challenges, and future directions for exosome-based therapies in OA treatment. Exosome-based therapies offer several distinct advantages over traditional OA treatments. Table 2 provides a comprehensive comparison of exosome-based therapy with traditional OA treatments, highlighting these key differences. In addition, Figure 3 visually represents this comparison, illustrating the unique benefits of exosome-based approaches.

Additionally, exosome-based therapies offer lower immunogenicity and better-targeted delivery compared with traditional treatments, enhancing their therapeutic efficacy and reducing potential side effects [27].

The mechanisms by which MSC-derived exosomes exert their therapeutic effects in OA are largely centered on their ability to mediate intercellular communication. As discussed in Section 2, exosomes are loaded with a diverse cargo of proteins, RNAs, and lipids, which allow them to modulate the activity of recipient cells. For instance, MSC-derived exosomes are enriched with growth factors, such as TGF-β and BMPs, which play a pivotal role in cartilage regeneration by promoting chondrocyte proliferation and ECM synthesis [16,28]. In addition, exosomes contain anti-inflammatory cytokines and miRNAs that inhibit the expression of matrix-degrading enzymes, such as MMP-13, a key contributor to cartilage degradation in OA [16,27]. This ability to suppress inflammation while promoting tissue repair makes exosomes an ideal candidate for OA therapy. The integration of exosome therapy with biomaterials represents another promising strategy for enhancing therapeutic efficacy, as highlighted in Section 4. The encapsulation of exosomes in biomaterials, such as hydrogels and scaffolds, allows for sustained and localized delivery to the site of injury. This improves the stability and bioavailability of exosomes and ensures a controlled release over time, which is critical for achieving long-lasting therapeutic effects in chronic conditions such as OA [28,30]. Hydrogels, for example, mimic the natural ECM and provide a favorable environment for exosomes, enhancing their retention at the injury site and facilitating the repair of damaged cartilage [30]. By integrating exosomes with these biomaterials, researchers can overcome some of the limitations associated with the direct administration of exosomes, such as rapid clearance from the joint cavity and short half-life.

However, despite the numerous advantages, the clinical application of exosome-based therapies for OA still faces several challenges, as discussed in Section 5. One of the primary challenges is the standardization of exosome isolation and characterization methods. Current isolation techniques, such as ultracentrifugation and immunoaffinity capture, are labor-intensive and time-consuming and vary in their ability to produce pure exosome preparations [27]. Variability in isolation methods can affect the quality and efficacy of exosome preparations, which poses a significant barrier to their clinical translation. To address this issue, standardized protocols for exosome isolation and purification need to be developed, ensuring that therapeutic exosomes meet consistent quality control standards across laboratories and clinical settings [31]. Scalability is another major hurdle for exosome therapies. Producing clinical-grade exosomes in sufficient quantities for widespread therapeutic use is a complex and resource-intensive process [26]. Current production methods typically yield low amounts of exosomes, which are insufficient to meet clinical demands. The development of scalable bioreactor systems, as mentioned in Section 5.2, holds promise for improving exosome yields and reducing the cost of production. Recent advances in bioengineering have shown that exosomes can be produced in larger quantities without compromising their therapeutic efficacy [52]. These efforts are essential for making exosome therapies more accessible and affordable for patients with OA.

From a regulatory perspective, the classification of exosomes as biological products or drug delivery systems presents unique challenges. As exosome therapies are relatively new, regulatory frameworks governing their production, clinical trials, and therapeutic use are still under development [47]. Regulatory agencies will need to establish guidelines for the characterization, safety, and efficacy of exosome-based therapies before they can be approved for widespread clinical use. In addition, long-term safety studies are crucial to evaluate the potential risks associated with exosome therapy, such as immunogenicity, tumorigenicity, and off-target effects [48,51,53]. Given that exosomes can modulate gene expression in recipient cells, there is a need for thorough investigations into the long-term consequences of altering cellular functions through exosome-mediated therapies [54]. Looking to the future, the therapeutic potential of MSC-derived exosomes in OA extends beyond their use as standalone treatments. Personalized medicine approaches, where exosome preparations are tailored to individual patients based on their specific genetic and clinical profiles, represent an exciting area of research [55]. By developing patient-specific exosome therapies, clinicians can deliver more targeted and effective treatments that address the unique needs of each individual. Additionally, combining exosome therapy with other regenerative approaches, such as gene editing and growth factor supplementation, may further enhance therapeutic outcomes in patients with OA [30,31,32,33,34].

## 8. Conclusions

Exosome-based therapies present a promising and innovative approach to treating OA as a whole-joint disease, targeting multiple joint tissues including cartilage, synovium, subchondral bone, and the infrapatellar fat pad. Unlike traditional therapies that primarily manage symptoms, exosomes offer regenerative capabilities by modulating the joint microenvironment, reducing inflammation, and promoting tissue repair across these interconnected tissues. Recent advancements in exosome isolation techniques, scalability through bioreactor systems, and bioengineering have enhanced the feasibility of producing clinical-grade exosomes. Additionally, progress in developing regulatory frameworks and standardizing production methods are paving the way for their clinical application. By addressing these challenges, exosome-based therapies are positioned to become a transformative solution for OA, providing a comprehensive and regenerative alternative that targets the disease’s underlying pathology. This approach offers the potential to not only alleviate symptoms but also restore joint function, improving patient outcomes and quality of life.

## Figures and Tables

**Table 1:** Comprehensive overview of exosome-based studies in osteoarthritis.

Author (year)	Title	Focus Area	Key Findings
Chen et al., 2023 [16]	The Application of Exosomes in Early Diagnosis and Treatment of Osteoarthritis	Exosome application in early OA diagnosis	Discussed how exosomes in the synovial fluid can serve as biomarkers and therapeutic agents for early-stage OA
He et al., 2020 [20]	Bone Marrow Mesenchymal Stem Cell-Derived Exosomes Protect Cartilage Damage	MSC-derived exosomes in OA	Demonstrated how BMSC-derived exosomes protect the cartilage from degeneration and reduce pain in OA models
Li et al., 2021 [21]	Exosomes Derived from Non-Classic Sources for Treatment of Post-Traumatic OA	Nonclassical exosome sources	Explored the use of exosomes from nontraditional sources, such as plant-derived exosomes, to treat post-traumatic OA
Cheng et al., 2022 [22]	Engineering of MSC-Derived Exosomes: A Promising Cell-Free Therapy for OA	MSC exosome engineering for cell-free therapy	Discussed MSC-derived exosome modifications to enhance therapeutic efficacy in OA treatment through improved targeting and potency
Tao et al., 2017 [25]	Exosomes from miR-140-5p Overexpressing Human Synovial MSCs Enhance Cartilage Tissue Regeneration	miR-140-5p in exosome-mediated therapy	Showed that exosomes enriched with miR-140-5p from MSCs can inhibit OA progression and promote cartilage regeneration
Duan et al., 2021 [27]	Exosome-Mediated Drug Delivery for Cell-Free Therapy of Osteoarthritis	Exosome-mediated drug delivery	Analyzed the effectiveness of exosome-mediated drug delivery systems as cell-free alternatives to traditional OA therapies
Chen et al., 2022 [28]	Biomaterials-Assisted Exosomes Therapy in Osteoarthritis	Biomaterials in exosome therapy	Investigated the role of biomaterials in enhancing exosome delivery, stability, and therapeutic efficacy for OA treatment
Maehara et al., 2021 [29]	Potential of Exosomes for Diagnosis and Treatment of Joint Disease	Exosome biology and therapeutic potential	Highlighted the diagnostic potential of exosomes in joint disease and their promising role in OA therapy
Wang et al., 2017 [30]	Exosomes from Embryonic MSCs Alleviate Osteoarthritis	ESC-derived exosomes in OA	Demonstrated how exosomes derived from embryonic stem cells reduce OA symptoms and aid cartilage repair in animal models
Cheng et al., 2024 [31]	Chondroprotective Effects of Bone Marrow MSC-Derived Exosomes in OA	MSC-derived exosomes in cartilage repair	Highlighted chondroprotective effects of MSC-derived exosomes and their ability to modulate inflammation and cartilage regeneration in OA
Luo et al., 2024 [32]	Mesenchymal Stem Cell-Derived Exosomes: A Cell-Free Therapy for Knee OA	MSC-derived exosomes and immune modulation	Focused on the immune-modulatory properties of exosomes and their therapeutic potential in OA treatment, particularly in reducing inflammation and modulating immune responses
Yang et al., 2024 [33]	Effects of Human Umbilical Cord MSC-Derived Exosomes in Rat OA Models	Umbilical cord MSC-derived exosomes in OA	Showed the therapeutic potential of umbilical cord-derived exosomes in animal models, emphasizing their ability to reduce OA-related inflammation and promote cartilage repair
Vadhan et al., 2024 [34]	MSC-Derived Exosomes as a Treatment Option for OA	MSC-derived exosomes for inflammation reduction	Examined MSC-derived exosomes’ potential to reduce inflammation and promote cartilage repair in OA

OA: osteoarthritis; MSC: mesenchymal stem cell; ESC: embryonic stem cells.

**Table 2:** Comparison of exosome-based therapy with traditional treatments for osteoarthritis.

Treatment Type	Mechanism of Action	Advantages	Limitations	Current Stage of Development/Use
NSAIDs	Inhibit cyclooxygenase enzymes, reduce inflammation	Easily accessible, effective for pain relief	Gastrointestinal side effects, cardiovascular risks with long-term use	Widely used, first-line treatment
Intra-articular corticosteroid injections	Suppress inflammation and pain	Rapid pain relief, can be repeated	Short-term effects, potential cartilage damage with repeated use	Commonly used in clinical practice
Hyaluronic acid injections	Improve joint lubrication and shock absorption	Improve joint function, longer-lasting effects than steroids	Variable efficacy, multiple injections needed	Approved and used clinically
MSC-based cell therapy	Differentiate into chondrocytes, secrete paracrine factors	Potential for cartilage regeneration, anti-inflammatory effects	Invasive, potential for immune rejection, variability in cell quality	Clinical trials ongoing, limited approved uses
Exosome-based therapy	Deliver bioactive molecules (miRNAs, proteins, lipids) to target cells	Cell-free, easier to store and handle, potentially more consistent than cell therapy	Still in early stages of research, optimal dosing and administration to be determined	Preclinical and early clinical trials

NASID: non-steroidal anti-inflammatory drug.

## Data Availability

No new data were created or analyzed in this study.
